# Supplementary material for: Doxazosin, a Classic Alpha 1-Adrenoceptor Antagonist, Overcomes Osimertinib Resistance in Cancer Cells via the Upregulation of Autophagy as Drug Repurposing
Source: Biomedicines. 2020 Aug 5;8(8):273. doi: 10.3390/biomedicines8080273 (PMC7460424; doi:10.3390/biomedicines8080273)
Supplement: Supplementary file 1 [file biomedicines-08-00273-s001.pdf]

Figure S1

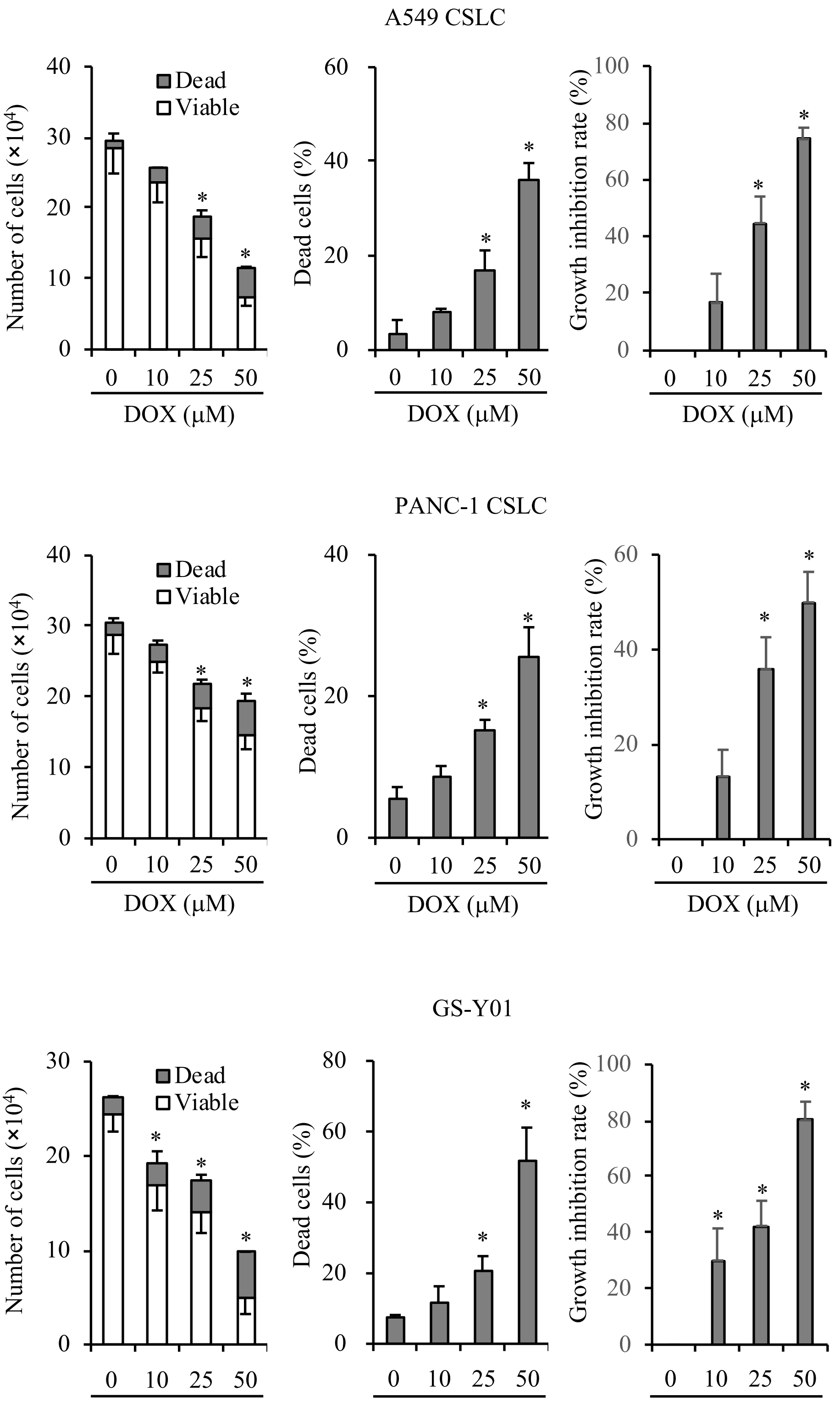

Figure S1. Doxazosin induces cancer stem cell death and inhibits cancer cell growth. Cells were treated with the indicated concentrations of doxazosin (DOX) for 3 days, and the numbers of viable and dead cells (left panels), the percentage of dead cells (center panels), and the growth inhibition rate (right panels) were assessed. Values in the graphs represent the means ± SD of triplicate samples of a representative experiment repeated with similar results. *: $p<0.05$.

Figure S2

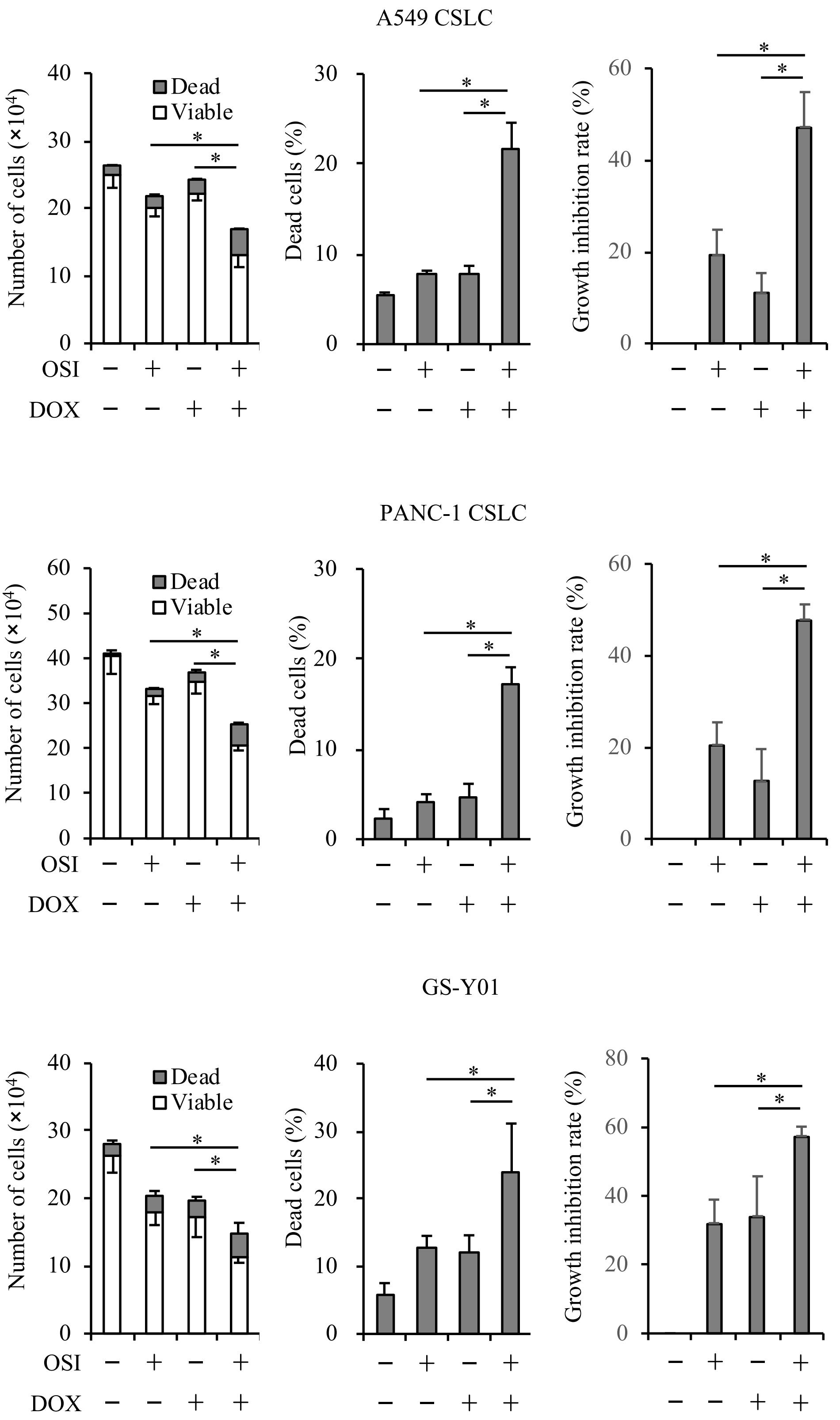

Figure S2. Doxazosin sensitizes several types of cancer stem cells to osimertinib. Cells were treated with/without 2 μM osimertinib (OSI), with/without 15 μM doxazosin (DOX) for 3 days, and the numbers of viable and dead cells (left panels) , the percentage of dead cells (center panels), and the growth inhibition rate (right panels) were assessed. Values represent the means ± SD of triplicate samples of a representative experiment repeated with similar results. *: $p<0.05$.

Figure S3

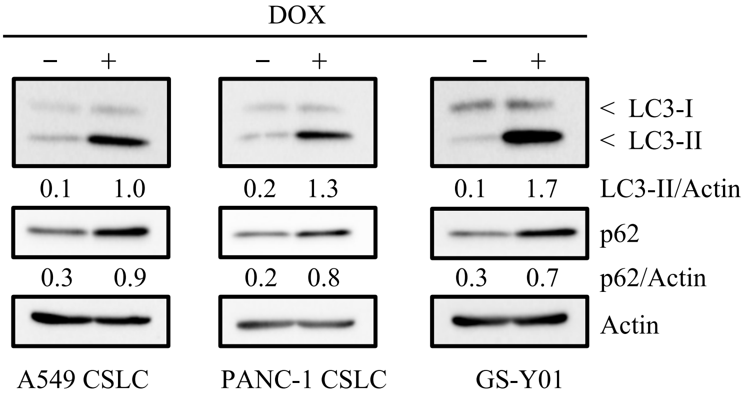

Figure S3. Doxazosin activates autophagy in cancer stem cells. Cells were treated with/without 15 μM doxazosin (DOX) for 3 days and then subjected to an immunoblot analysis for autophagic markers. The relative density was shown below each band of immunoblot.

Figure S4

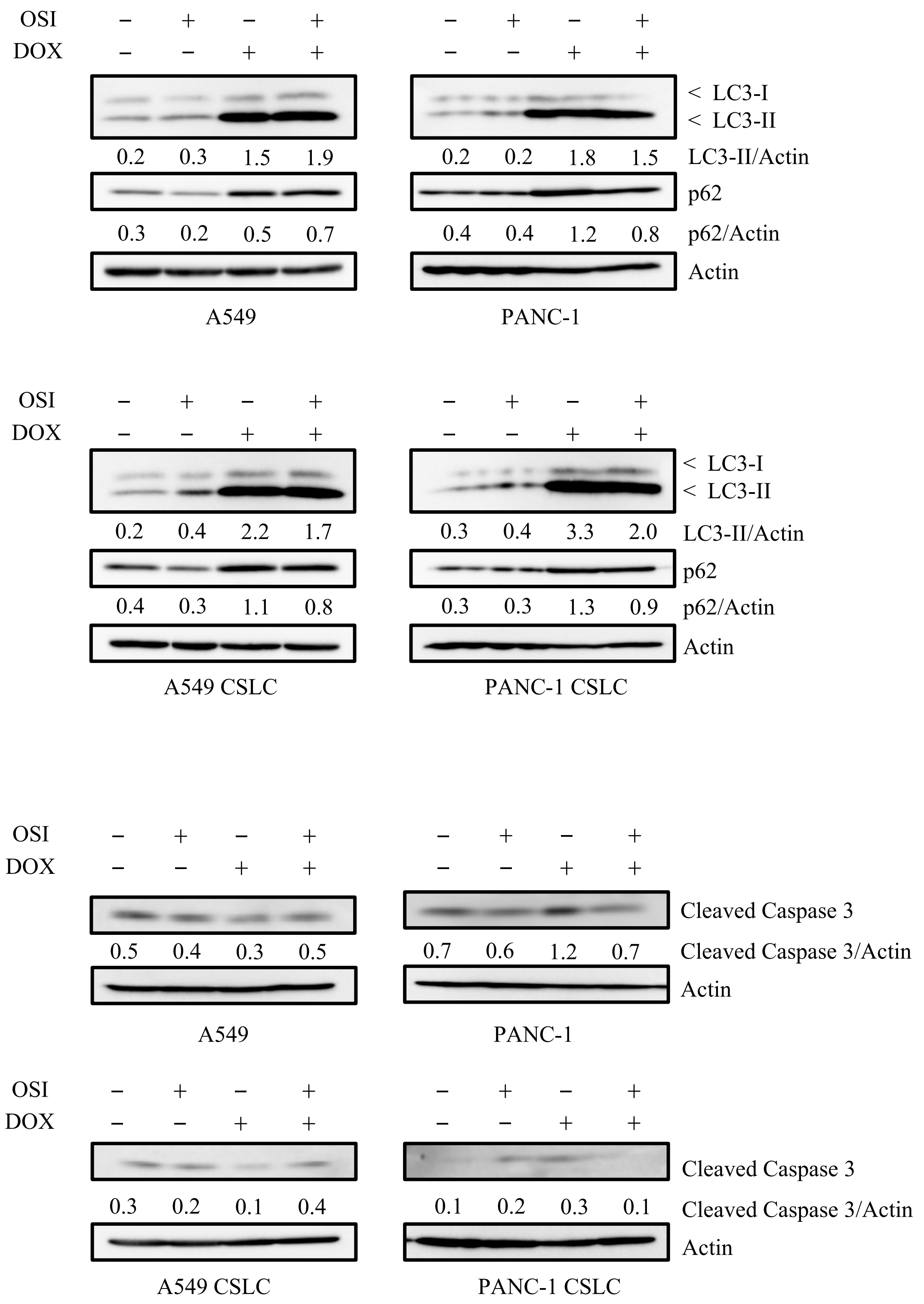

Figure S4. Effect of doxazosin and osimertinib on autophagy and apoptosis. A549, PANC-1, A549 CSLC, and PANC-1 CSLC cells were treated with/without 2 μM osimertinib (OSI) or with/without 15 μM doxazosin (DOX) for 3 days, and then subjected to an immunoblot analysis for autophagic markers and cleaved caspase 3. The relative density was shown below each band of immunoblot.
